# Effects of gut microbiota on omega-3-mediated ovary and metabolic benefits in polycystic ovary syndrome mice

**DOI:** 10.1186/s13048-023-01227-w

**Published:** 2023-07-14

**Authors:** Hong Zhang, Lu Zheng, Chuwei Li, Jun Jing, Zhou Li, Shanshan Sun, Tongmin Xue, Kemei Zhang, Mengqi Xue, Chun Cao, Lei Ouyang, Zhang Qian, Rui Xu, Zhaowanyue He, Rujun Ma, Li Chen, Bing Yao

**Affiliations:** 1 Center of Reproductive Medicine, Jinling Hospital, Affiliated Hospital of Medical School, Nanjing University, 305 Zhongshan East Road, Jiangsu 210002 Nanjing, China; 2 Center of Reproductive Medicine, Nanjing Jinling Hospital, School of Medicine, Jiangsu University, 212000 Zhenjiang, China; 3 Jiangsu Key Laboratory for Molecular and Medical Biotechnology, College of Life Sciences, Nanjing Normal University, 1 Wen Yuan Road, Jiangsu 210023 Nanjing, China; 4 Reproductive Medical Center, Clinical Medical College (Northern Jiangsu People’s Hospital), Yangzhou University, 98 Nantong West Road, Jiangsu 225001 Yangzhou, China; 5 Reproductive Medical Center, Jinling Hospital Department, Nanjing Medical University, 305 Zhongshan East Road, Jiangsu 210002 Nanjing, China; 6 Department of Reproductive Medicine, Affiliated Jinling Hospital, The First School of Clinical Medicine, Southern Medical University, 210002 Nanjing, China

**Keywords:** Omega-3 PUFAs, Polycystic ovary syndrome, Inflammation, Gut microbiota

## Abstract

**Background:**

Polycystic ovary syndrome (PCOS) is a common reproductive endocrine disorder that frequently exhibits low-grade inflammation, pro-oxidant activity, and gut dysbiosis. PCOS has become one of the leading causes of female infertility worldwide. Recently, omega-3 polyunsaturated fatty acids (PUFAs) have been proven to benefit metabolic disorders in PCOS patients. However, its roles in the regulation of metabolic and endocrinal balances in PCOS pathophysiology are not clear. In the present study, we aimed to explore how omega-3 PUFAs alleviate ovarian dysfunction and insulin resistance in mice with dehydroepiandrosterone (DHEA)-induced PCOS by modulating the gut microbiota.

**Methods:**

We induced PCOS in female mice by injecting them with DHEA and then treated them with omega-3 PUFAs. 16S ribosomal DNA (rDNA) amplicon sequencing, fecal microbiota transplantation (FMT) and antibiotic treatment were used to evaluate the role of microbiota in the regulation of ovarian functions and insulin resistance (IR) by omega-3 PUFAs. To further investigate the mechanism of gut microbiota on omega-3-mediated ovarian and metabolic protective effects, inflammatory and oxidative stress markers in ovaries and thermogenic markers in subcutaneous and brown adipose tissues were investigated.

**Results:**

We found that oral supplementation with omega-3 PUFAs ameliorates the PCOS phenotype. 16S rDNA analysis revealed that omega-3 PUFA treatment increased the abundance of beneficial bacteria in the gut, thereby alleviating DHEA-induced gut dysbiosis. Antibiotic treatment and FMT experiments further demonstrated that the mechanisms underlying omega-3 benefits likely involve direct effects on the ovary to inhibit inflammatory cytokines such as IL-1β, TNF-α and IL-18. In addition, the gut microbiota played a key role in the improvement of adipose tissue morphology and function by decreasing multilocular cells and thermogenic markers such as Ucp1, Pgc1a, Cited and Cox8b within the subcutaneous adipose tissues.

**Conclusion:**

These findings indicate that omega-3 PUFAs ameliorate androgen-induced gut microbiota dysbiosis. The gut microbiota plays a key role in the regulation of omega-3-mediated IR protective effects in polycystic ovary syndrome mice. Moreover, omega-3 PUFA-regulated improvements in the ovarian dysfunction associated with PCOS likely involve direct effects on the ovary to inhibit inflammation. Our findings suggest that omega-3 supplementation may be a promising therapeutic approach for the treatment of PCOS by modulating gut microbiota and alleviating ovarian dysfunction and insulin resistance.

**Supplementary Information:**

The online version contains supplementary material available at 10.1186/s13048-023-01227-w.

## Introduction

Polycystic ovary syndrome (PCOS) is a common endocrine and metabolic disorder that affects 6–10% of women of reproductive age [1, 2]. PCOS is characterized by clinical features including androgen excess irregular menses, ovulatory dysfunction and polycystic ovarian morphology [3]. Additionally, PCOS is often accompanied by metabolic disorders such as insulin resistance (IR), hyperandrogenemia, hyperinsulinemia and obesity [4, 5]. Moreover, subsequent morbidities, such as type 2 diabetes mellitus (T2DM), cardiovascular disease, endometrial cancer and psychiatric conditions, exert much influence on individuals with PCOS [6–8]. Infertility and metabolic syndrome are major concerns of women with PCOS [9]. The proposed mechanisms underlying PCOS vary from genetic, lifestyle, and environmental triggers, but the pathogenesis of PCOS is poorly understood [10–12]. Meanwhile, evidence has shown that hyperandrogenemia, inflammation, and oxidative damage play a key role in the pathophysiology of PCOS [13]. PCOS females have significantly increased levels of proinflammatory factors, such as tumor necrosis factor-α (TNF-α), interleukin-1β (IL-1β), IL- 18 and monocyte chemotactic protein (MCP-1) [14]. To date, there is no approved pharmacologic therapy for PCOS because the pathogenesis of PCOS is poorly understood and there is no consensus on the underlying mechanisms, and most drugs, such as oral contraceptives, antiandrogens, insulin sensitizers, and aromatase inhibitors, are used to treat the symptoms of PCOS in an off-label manner [15]. Lifestyle modifications and weight loss remain significant therapies for PCOS. Recently, nutrition interventions such as low-glycemic index diets and Mediterranean diets have become recommended treatments for women with PCOS [16–19].

Nutritional intervention for women with PCOS has attracted attention. In recent years, studies have shown that dietary fat is considered a biological regulator affecting metabolism, and omega-3 PUFAs may be the most effective metabolic fatty acid regulator [20–23]. Animal studies have shown that omega-3 PUFAs can prevent IR by reducing inflammation and depositing fat in insulin-sensitive tissues [24]. In addition, omega-3 PUFAs can positively affect fertility, as they play an essential role in steroidogenesis [25]. However, the role and mechanism of omega-3 fatty acid supplementation in PCOS is still uncertain. Therefore, we designed a DHEA-induced mouse model to investigate the effects of supplementation with omega-3 PUFAs on PCOS mice.

Increasing evidence shows that alterations in the gut microbiome have specific impacts on every stage of female reproduction, including follicle and oocyte maturation in the ovary, fertilization and embryo migration [26–28]. Recent studies have shown that PCOS is inextricably linked to the gut microbiota, and individuals with PCOS have gut microbiota communities different from those of healthy controls [29–31]. It has been proposed that IR, metabolic abnormalities, and sex hormone concentrations in women with PCOS may affect the diversity and composition of the gut microbiota. Moreover, gut microbiota dysbiosis could contribute to IR and obesity in patients with PCOS because of the stimulation of inflammatory activities and disruption of energy balance [32, 33]. The restoration of gut microbiota dysbiosis through dietary probiotic inulin supplementation or α-linolenic acid-rich flaxseed oil contributes to the amelioration of PCOS [34]. Accumulating evidence suggests a correlation between omega-3 PUFAs and the gut microbiota. Omega-3 PUFAs protect against intestinal barrier and permeability dysfunctions by increasing healthy bacterial composition and anti-inflammatory compounds such as short-chain fatty acids (SCFAs) while inhibiting the production of pro-inflammatory mediators such as lipopolysaccharide (LPS) [35]. Omega-3 PUFAs can increase the abundance of lactic acid-producing bacteria and decrease the *Firmicutes*-to-*Bacteroidetes* (*F*/*B*) ratio in high-fat diet-fed mice [36]. However, the effect of omega-3 PUFAs on the gut microbiota in PCOS models has not been investigated. Considering the regulatory effect of omega-3 PUFAs on the gut microbiota, associations among omega-3 PUFAs, the gut microbiota and PCOS deserve investigation. Future studies could investigate whether omega-3 PUFAs directly modulate gut microbial composition and function and whether this modulation has therapeutic effects in PCOS models.

In the present study, we aimed to explore how omega-3 PUFAs alleviate ovarian dysfunction and IR in mice with DHEA-induced PCOS by modulating the gut microbiota. We investigated the regulation of omega-3 PUFAs on gut microbial composition in DHEA-induced PCOS mouse models. We report that omega-3 PUFAs attenuate IR in PCOS in a gut microbiota-dependent manner. Moreover, omega-3 PUFA-regulated improvements in the ovarian dysfunction associated with PCOS likely involve direct effects on the ovary to inhibit inflammation. We show that omega-3 PUFAs represent potential prebiotic agents for the treatment of PCOS.

## Methods

### Animals and treatments

Female (3 weeks old) C57BL/6 mice were purchased from Jiangsu ALF Biotechnology Company and housed in specific pathogen-free (SPF) conditions with free access to regular rodent chow and water. The SPF animal facility, including germ-free isolators at Jinling Hospital, was maintained in a 12-hour light and dark cycle at 23 ± 2 °C and 50 ± 10% humidity. Use a microisolator (CBC filter bonneted) and individually ventilated cages (PIV/IVC). Sterilize or disinfect food, water, cages and anything that will come in contact with the mice. The cage and water were changed twice a week. All animal protocols were approved by the Animal Care and Use Committee of Jinling Hospital. All of the experiments were carried out in accordance with the approved guidelines by Jinling Hospital. They were randomly divided into different groups and housed in individually ventilated cages (3–5 mice in each cage).

The DHEA-induced PCOS mouse model was established based on a previous report [37]. Mice in the experimental groups were subcutaneously injected with DHEA dissolved in sesame oil (D4000; Sigma–Aldrich; 60 mg/kg body weight per day for 21 days, *n* = 6 each).

For the omega-3-treated mice, after 3 weeks of DHEA treatment, the mice received another 8 weeks of treatment by gavage with omega-3 PUFAs (2 g/kg every 2 days) for the omega-3 group (*n* = 6 each), while PCOS mice were treated with an equal volume of corn oil at 2 g/kg for comparison. The mice were anesthetized with ether and killed by cervical dislocation. Cervical dislocation after anesthesia is a humane method of euthanasia according to the Guide for the Care and Use of Laboratory Animals (Eighth Edition) made by the Institutional Animal Care & Use Committee.

Serum was collected to measure sex hormone levels at diestrus stages. The ovaries were processed for hematoxylin and eosin staining. The remaining ovaries were quickly frozen and stored at − 80 °C. For H&E staining, ovaries were fixed in 4% paraformaldehyde (PFA) for 24 h and embedded in paraffin. Section (5 μm) were prepared and dewaxed prior to straining. The sera were collected for hormone level analysis; ovarian tissues were collected for H&E staining and real-time quantitative PCR analysis.

### Vaginal smears and estrous cycle determination

Vaginal smears were taken daily at 9:00 a.m. from the 75th to the 84th day after the first day of treatment. The stage of the estrous cycle was determined by microscopic analysis of the predominant cell type in vaginal smears following H&E staining. Proestrus consists of round, nucleated epithelial cells; estrus consists of cornified squamous epithelial cells; metestrus consists of epithelial cells and leukocytes; and diestrus consists of nucleated epithelial cells and a predominance of leukocytes. The estrous cycle was determined by classic criteria [38].

### Serum analysis

Blood samples were collected in 1.5 mL EP tubes, blood clotting was performed for 1 h at 4 °C, and the tubes were centrifuged at 500 × g/4°C for 10 min to harvest serum. Serum samples and conditional cell culture media were stored at -80 °C for subsequent serum determinations. The levels of follicle-stimulating hormone (FSH) (CEA830Mu; Cloud-Clone), luteinizing hormone (LH) (CEA441Mu; Cloud-Clone), estradiol 2 (E2) (ml001962; mlbio) and testosterone (T) (ml001954; mlbio) were determined by enzyme-linked immunosorbent assay kits for mice.

### RNA extraction and real-time quantitative PCR (qPCR)

Total RNA was extracted using a Total RNA Purification Kit (B0004-plus; EZBioscience), and cDNA was reverse-transcribed from 1 µg total RNA using HiScript III RT SuperMix (R312-01; Vazyme) according to the manufacturer’s protocol. RT–qPCR was performed in triplicate in 96-well plates using SYBR qPCR Master Mix (Q111-02; Vazyme) with gene-specific forward and reverse primers. cDNA was amplified for 40 cycles using a Roche LC480 Real-time PCR system. *β-actin* was used as a reference gene. The qPCR primers used in this study can be found in Table 1.

**Table 1:** Primer sequences for RT-qPCR (from 5’ to 3’ terminal)

Genes	Forward	Reverse
*β-actin*	5’-GGCTGTATTCCCCTCCATCG-3’	5’- CCAGTTGGTAACAATGCCATGT-3’
*Ucp1*	5’-AGGCTTCCAGTACCATTAGGT-3’	5’-CTGAGTGAGGCAAAGCTGATTT-3’
*Pgc1a*	5’-TATGGAGTGACATAGAGTGTGCT-3’	5’-CCACTTCAATCCACCCAGAAAG-3’
*Cited1*	5’-AACCTTGGAGTGAAGGATCGC-3’	5’-GTAGGAGAGCCTATTGGAGATGT-3’
*Cox8b*	5’-TGTGGGGATCTCAGCCATAGT-3’	5’-AGTGGGCTAAGACCCATCCTG-3’
*Il1b*	5’- ATGATGGCTTATTACAGTGGCAA-3’	5’- GTCGGAGATTCGTAGCTGGA-3’
*Il18*	5’- TCTTCATTGACCAAGGAAATCGG-3’	5’- TCCGGGGTGCATTATCTCTAC-3’
*Tnfa*	5’- GAGGCCAAGCCCTGGTATG-3’	5’- CGGGCCGATTGATCTCAGC-3’
*Ccl2*	5’- TTAAAAACCTGGATCGGAACCAA-3’	5’-GCATTAGCTTCAGATTTACGGGT-3’

### 16S rDNA amplicon sequencing and bioinformatics analysis

Microbial DNA was extracted using HiPure StoolDNA Kits (Magen, Guangzhou, China) according to the manufacturer’s protocols. High-throughput sequencing was used to research the V3 − V4 region of 16S rDNA with a sequencing depth of 60,000 in the control, PCOS and omega-3 groups (*n* = 3 each). The 16S rDNA V3-V4 region of the ribosomal RNA gene was amplified by PCR (94 °C for 2 min, followed by 30 cycles at 98 °C for 10 s, 62 °C for 30 s, and 68 °C for 30 s and a final extension at 68 °C for 5 min) using primers 341 F: CCTACGGGNGGCWGCAG; 806R: GGACTACHVGGGTATCTAAT. PCRs were performed in triplicate in a 50 µL mixture containing 5 µL of 10. KOD buffer, 5 µL of 2 mM dNTPs, 3 µL of 25 mM MgSO4, 1.5 µL of each primer (10 µM), 1 µL of KOD polymerase, and 100 ng of template DNA. Noisy sequences of raw tags were filtered by the QIIME (version 1.9.1) pipeline under specific filtering conditions to obtain high-quality clean tags. Clean tags were searched against the reference database (version r20110519) to perform reference-based chimera checking using the UCHIME algorithm. All chimeric tags were removed, and finally, the obtained effective tags were used for further analysis. The effective tags were clustered into operational taxonomic units (OTUs) of ≥ 97% similarity using the UPARSE (version 9.2.64) pipeline. Taxonomic classification was conducted by BLAST (version 2.6.0), searching the representative OTU sequences against the NCBI 16S ribosomal RNA Database (Bacteria and archaea) (http://www.ncbi.nlm.nih.gov) (version 202,101) using the best hit with strict criteria (E value < e-5, query coverage ≥ 60% and the following identity thresholds: a hit with sequence identity ≥ 92% was considered to belong to the same species; with sequence identity ≥ 88% as indicators of belonging to the same genus; with sequence identity ≥ 85% as indicators of the same family; with sequence identity ≥ 80% as indicators of the same order; the classes were inferred when sequence identity ≥ 75%; the phylum were inferred when sequence identity ≥ 70%). The tag sequence with the highest abundance was selected as a representative sequence within each cluster. The representative sequences were classified into organisms by a naive Bayesian model using the RDP classifier (version 2.2) with confidence threshold values ranging from 0.8 to 1.

We evaluated the adequacy of sequencing by plotting the rarefaction curve for each index and indirectly reflected the richness of species in the sample. The rarefaction curve calculates the expected value of each index by sampling n tags several times and then makes a curve according to a set of n values (i.e., the number of samplings, which is generally a group of arithmetic sequences less than the total number of sequences) and the corresponding expected value of the index. When the curve flattens or reaches a plateau, it can be considered that the increase in sequencing depth has no effect on species diversity. This indicated that the amount of sequencing was sufficient. The OTU rarefaction curve was plotted in the R project ggplot2 package (version 2.2.1).

Sequences were rarefied prior to calculation of alpha and beta diversity statistics. Alpha diversity indexes were calculated in QIIME (version 1.9.1) from rarefied samples using the Shannon index for diversity and the Chao1 index for richness. Beta diversity was calculated using the unweighted pair group method using the arithmetic average (UPGMA) clustering method and principal component analysis (PCA). The total OTUs are shown in Supplementary Table 1.

### Antibiotic treatment and fecal microbiota transplantation

The mice were treated with antibiotics for 7 d before transplantation experiments. Six-week-old PCOS C57BL/6 mice were supplemented with either omega-3 PUFAs or an equal volume of corn oil at 2 g/kg in the presence of the antibiotic vancomycin (0.5 g/liter), neomycin sulfate (1 g/liter), metronidazole (1 g/liter), and ampicillin (1 g/liter) [39].

Briefly, 6-week-old female donor PCOS mice were treated with omega-3 PUFAs or an equal volume of corn oil (2 g/kg). The recipient mice were treated with fresh transplant material from either PCOS mice or omega-3 PUFA-treated mice. Stool was collected daily from donor mice and pooled. Donor stool (100 mg) was diluted with saline, homogenized for 1 min using a vortex to achieve a liquid slurry, and then centrifuged at 500 × g for 3 min to remove particulate matter to facilitate administration. A total of 100 µl of the fecal suspension from either PCOS mice or omega-3 PUFA-treated mice was administered to mice by oral gavage. Fresh transplant material was prepared within 10 min before oral gavage to prevent changes in bacterial composition. Oral gavage with fecal transplant material was conducted every 2 days throughout the 8-week experiment.

### Statistical analysis

Statistical analysis was performed using GraphPad Prism version 8 for Windows. The normality of the data was analyzed first, and then statistical tests were performed. Student’s t test, the Mann–Whitney test, and one-way analysis of variance (ANOVA) followed by Tukey’s post hoc test were used. All other data are presented as the mean ± standard error of the mean. The statistical significance threshold: alpha = 0.05 for hypothesis testing, as *p* value < alpha, we conclude that the null hypothesis should be rejected. And *p* values are denoted in figures and indicate the following: *P < 0.05; **P < 0.01; ***P < 0.001, ****P < 0.0001.

## Results

### Omega-3 PUFA supplementation attenuates endocrine and metabolic disorders in DHEA-induced PCOS mice

**Fig. 1:**
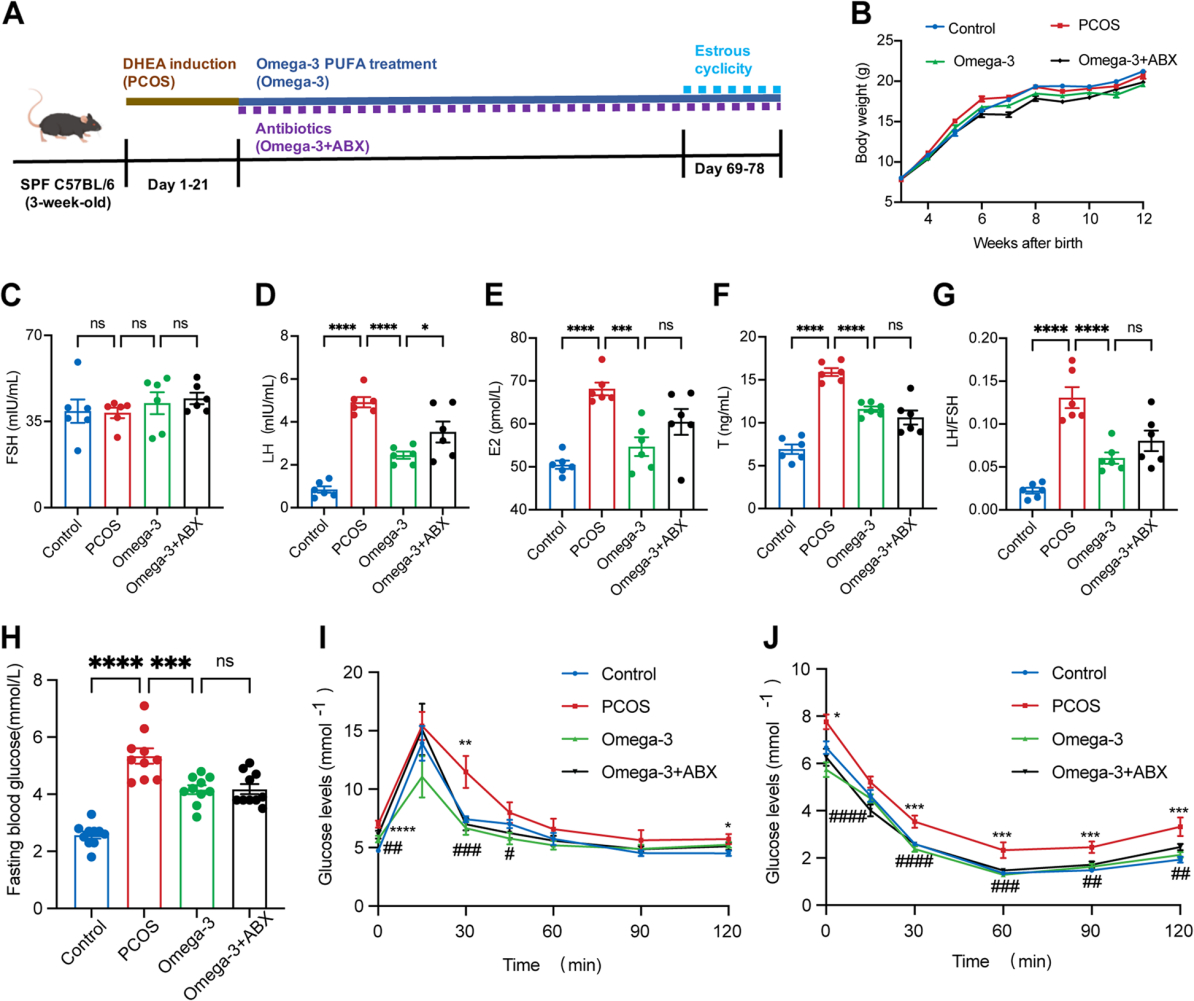
Omega-3 PUFA supplementation attenuates metabolic and endocrine disorders in DHEA-induced PCOS mice. **A** Experimental design. **B** Body weights (*n* = 6). **C**-**G** The serum levels of FSH (**C**), LH (**D**), E2 (**E**), T (**F**) and LH/FSH (**G**) were measured by ELISA (*n* = 6). **H** Fasting blood glucose (*n* = 10). **I** GTT (*n* = 10). **J** ITT (*n* = 10), * represents the control group compared to the PCOS group, and # represents PCOS mice compared to the omega-3 group. The data are shown as the mean ± SEM with individual values. Statistical analyses were carried out using one-way ANOVA followed by Dunnett’s multiple comparisons test (*P < 0.05; **P < 0.01; ***P < 0.001; ****P < 0.0001). FSH, follicle-stimulating hormone; LH, luteinizing hormone; E2, estradiol 2; T, testosterone; GTT, glucose tolerance test; ITT, insulin tolerance test

Because PCOS is closely associated with endocrine and metabolic disorders, to investigate the effect of omega-3 PUFAs on metabolism and endocrine function, we established a PCOS model (Fig. 1A) and assessed serum hormone levels, glucose homeostasis and insulin sensitivity in diverse groups (Fig. 1). During the 8-week treatment, the body weight of the PCOS mice was similar to that of the control group. In comparison with mice treated with DHEA alone, omega-3 PUFA treatment did not change body weight (Fig. 1B). No differences were observed in FSH levels among the three groups (Fig. 1C). The levels of LH, E2, T and the LH/FSH ratio were elevated in the PCOS mice, but these effects were reversed by omega-3 PUFA treatment (Fig. 1D-G). In addition, compared with control mice, the PCOS mice displayed higher fasting blood glucose levels, in which omega-3 PUFAs significantly decreased (Fig. 1H). Compared with healthy controls, mice treated with DHEA displayed insulin resistance, as revealed by glucose tolerance tests (GTTs) and insulin tolerance tests (ITTs). This impairment in insulin resistance was improved by omega-3 PUFA treatment (Fig. 1I, J). Together, these results demonstrate that omega-3 PUFA supplementation improved insulin resistance and sex hormone disorders in DHEA-induced PCOS mice.

### Omega-3 PUFAs attenuate ovarian dysfunction in DHEA-induced PCOS mice

**Fig. 2:**
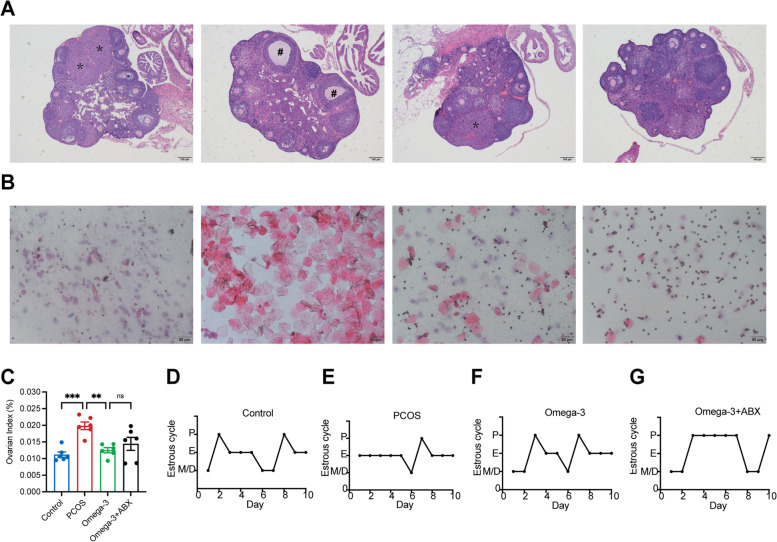
Omega-3 PUFAs attenuate ovarian dysfunction in DHEA-induced PCOS mice. **A** Representative images of ovarian sections. Scale bars: 100 μm. The asterisk (*) represents the corpus luteum, and the pound sign (#) represents the ovary vacuoles. **B** Estrous cycle changes. **C** Ovarian index. **D**-**G** Representative estrous cycles in representative mice from diverse groups. The data are shown as the mean ± SEM with individual values. Statistical analyses were carried out using one-way ANOVA followed by Dunnett’s multiple comparisons test (**P < 0.01; ***P < 0.001). P, proestrus stage; E, estrus stage; M, metestrus stage; D, diestrus stage.

Infertility and metabolic syndrome are major concerns of women with PCOS. Next, we measured the effects of omega-3 PUFAs on ovarian function in PCOS-like mouse models. H&E staining was used to determine the alteration of ovarian pathology in diverse groups (Fig. 2A). The PCOS group exhibited a higher number of cystic follicles and a lower number of corpus lutea than the control group. On the other hand, omega-3 PUFA supplementation decreased the number of cystic follicles and increased the formation of corpus lutea (Fig. 2A). Compared with the PCOS group, omega-3 PUFA-treated mice displayed a decreased ovarian index and reversed disrupted estrous cycles (Fig. 2B-G). Together, these results suggest that omega-3 PUFAs ameliorated ovarian dysfunction in PCOS mice.

### Omega-3 PUFAs alleviate gut dysbiosis in PCOS mice

**Fig. 3:**
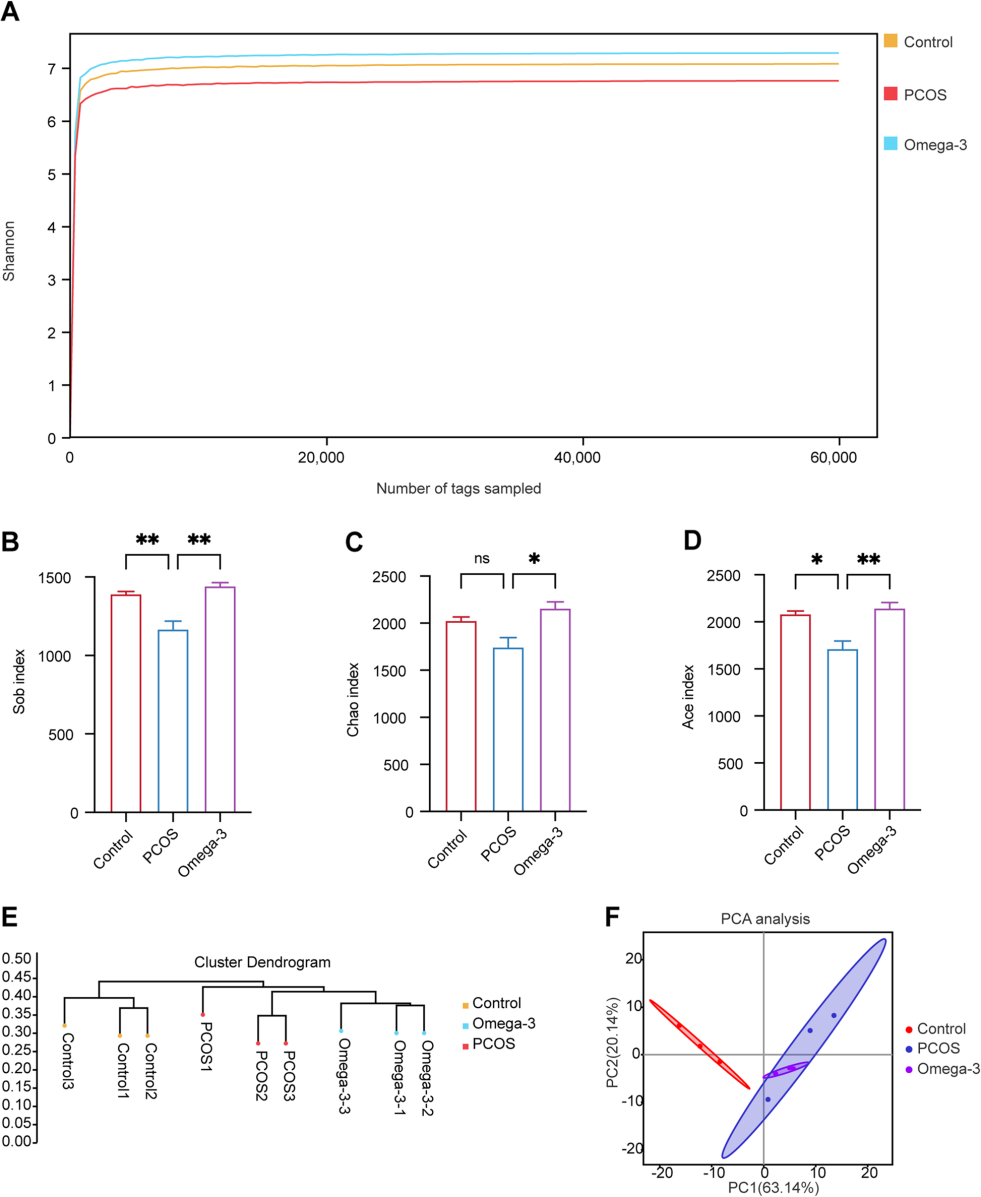
Omega-3 PUFA treatment has an obvious effect on the α-diversity but not β-diversity of the gut microbiota in PCOS mice. The microbiota composition of control, PCOS and omega-3 mice was analyzed by 16 S rDNA pyrosequencing (*n* = 3 for each group). **A** Shannon curve. **B** Sob index. **C** Chao1 index. **D** Ace index. **E** UPGMA clustering method. **F** PCA of gut microbiota based on the OTU data of the control, PCOS, and omega-3 groups. PCA, principal component analysis; UPGMA, unweighted pair group method using arithmetic average. The data are shown as the mean ± SEM with individual values. Statistical analyses were carried out using one-way ANOVA followed by Dunnett’s multiple comparisons test (*P < 0.05; **P < 0.01)

To explore the effects of omega-3 PUFAs on gut microbiota, we harvested fecal samples from the control, PCOS and omega-3 PUFA-treated mice and then performed 16 S rDNA analysis. Subsequently, the abundance and diversity of the bacterial community were assessed by the Shannon curve (Fig. 3A) and three α-diversity indexes, the Sob index, Chao 1 index and abundance-based coverage estimator (ACE) index. The Sob index, Chao1 and Ace index of the gut microbiota were significantly altered by omega-3 PUFAs (Fig. 3B-D), which indicates that omega-3 PUFA treatment has an obvious effect on the α-diversity of gut microbiota in PCOS mice. Next, we used the UPGMA clustering method and PCA to assess the β-diversity of the gut microbiota across diverse groups. The UPGMA clustering method showed that omega-3 PUFA supplementation affected the gut microbial profile of the PCOS mice (Fig. 3E). However, the distinction of PCA was not much different between the PCOS and omega-3 groups (Fig. 3F). Together, these results suggest that the β-diversity of the gut microbiota is not affected by omega-3 treatment.

**Fig. 4:**
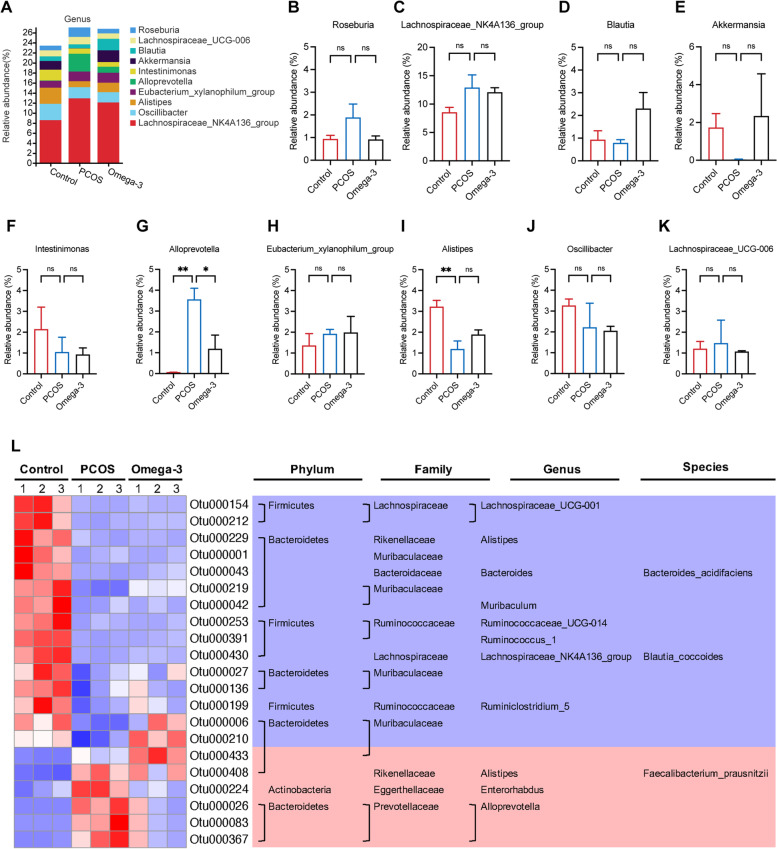
Omega-3 PUFAs modulate the gut microbiota of PCOS mice. **A** Bacterial taxonomic profiling at the genus level of intestinal bacteria from different mouse groups. **B**-**K** Relative abundance of *Lachnospiraceae_NK4A136_group*, *Oscillibacter*, *Alistipes*, *Eubacterium_xylanophilum_group*, *Alloprevotella*, *Intestinimonas*, *Akkermansia*, *Blautia*, *Lachnospiraceae_UCG-006*, and *Roseburia*. **L** Heatmap of the 21 OTUs in the control group altered in PCOS in response to omega-3 PUFA treatment. The phylum, family, and genus names of the OTUs are shown in the right panel. The color of the spots in the left panel represents the relative abundance at the genus level in each group. The analyses were conducted using R software version 3.3.1

To assess the overall composition of the bacterial community in different groups, we analyzed the degree of bacterial taxonomic similarity at the genus level (Fig. 4A). Compared to control mice, PCOS mice displayed an increase in the relative abundance of *Alloprevotella* and a decrease in the relative abundance of *Akkermansia* and *Alistipes*, while omega-3 PUFA treatment protected against these effects (Fig. 4B-K). A heatmap was further used to identify the specific bacterial phylotypes that were altered by omega-3 PUFAs (Fig. 4L). In control mice, treatment with DHEA significantly increased 6 operational taxonomic units (OTUs) and decreased 15 OTUs (Fig. 4L). Among these 21 OTUs, omega-3 PUFA treatment regulated 20 OTUs, which were altered in PCOS mice compared with control mice (Fig. 4L). These results indicate that omega-3 PUFAs modulated the gut microbiota of PCOS mice.

### Gut microbiota mediates part of the beneficial effects of omega-3 PUFAs on the ovaries

**Fig. 5:**
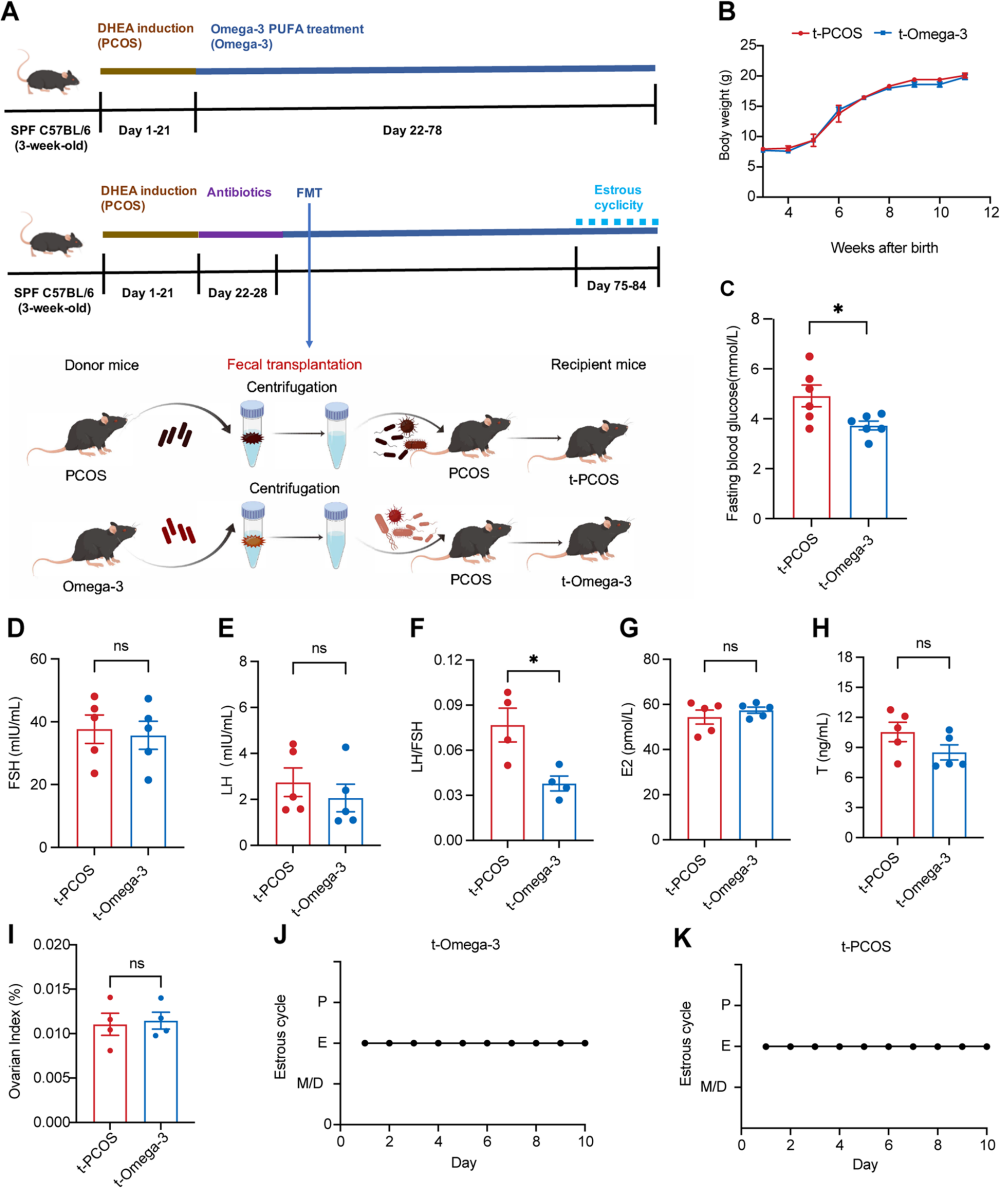
Gut microbiota mediates part of the beneficial effects of omega-3 PUFAs on the ovaries. Mice were randomly divided into four groups. PCOS mice were orally administered corn oil (PCOS) or omega-3 PUFAs (2 g/kg every 2 days). Horizontal fecal transfer from saline–treated PCOS mice is referred to as PCOS receivers (PCOS→PCOS). Horizontal fecal transfer from omega-3 PUFA-treated mice is referred to as omega-3 receivers (omega-3→PCOS). **A** Study design of the fecal transplantation experiment. **B** Body weight of the above four groups of mice. **C** Fasting blood glucose (*n* = 6). **D**-**H** Serum FSH, LH, LH/FSH ratio, E2 and T levels (*n* = 5). **I** Ovarian index (*n* = 4). **J**, **K** Representative estrous cycles. Error bars are expressed as the means ± SEMs. Statistical significance was determined by *P* values determined by two-tailed paired-samples t test (*P < 0.05). P, proestrus stage; E, estrus stage; M, metestrus stage; D, diestrus stage; FSH, follicle-stimulating hormone; LH, luteinizing hormone; E2, estradiol 2; T, testosterone

To further investigate whether the beneficial effects of omega-3 PUFAs were mediated by the gut microbiota, we transferred the microbiota from omega-3 PUFA-treated mice to PCOS recipient mice (Fig. 5A). After 8 weeks of colonization, horizontal fecal transfer from omega-3 PUFA-treated mice (omega-3→PCOS) demonstrated some protective effects similar to those observed in the omega-3 PUFA-treated group, such as fasting blood glucose (Fig. 5C) and LH/FSH ratio (Fig. 5F). However, the body weight (Fig. 5B), ovarian index, serum FSH, LH, E2 and T levels were not significantly different between the t-PCOS and t-Omega-3 groups (Fig. 5D-I). Compared with t-PCOS mice, t-Omega-3 mice also displayed disrupted estrous cycles (Fig. 5J, K). To further confirm whether the protective effects of omega-3 were conducted by microbes, we treated omega-3 PUFA-treated PCOS mice with a cocktail of antibiotics, which included vancomycin, neomycin sulfate, metronidazole, and ampicillin. When the gut microbiota was suppressed by the antibiotic cocktail, only the protective effects of LH levels were abolished (Fig. 1). Collectively, these results demonstrated that the gut microbiota mediates part of the beneficial effects of omega-3 PUFAs on the ovaries.

### Omega-3 PUFAs improve the function and morphology of adipose tissues in PCOS in a gut microbiota–dependent manner

**Fig. 6:**
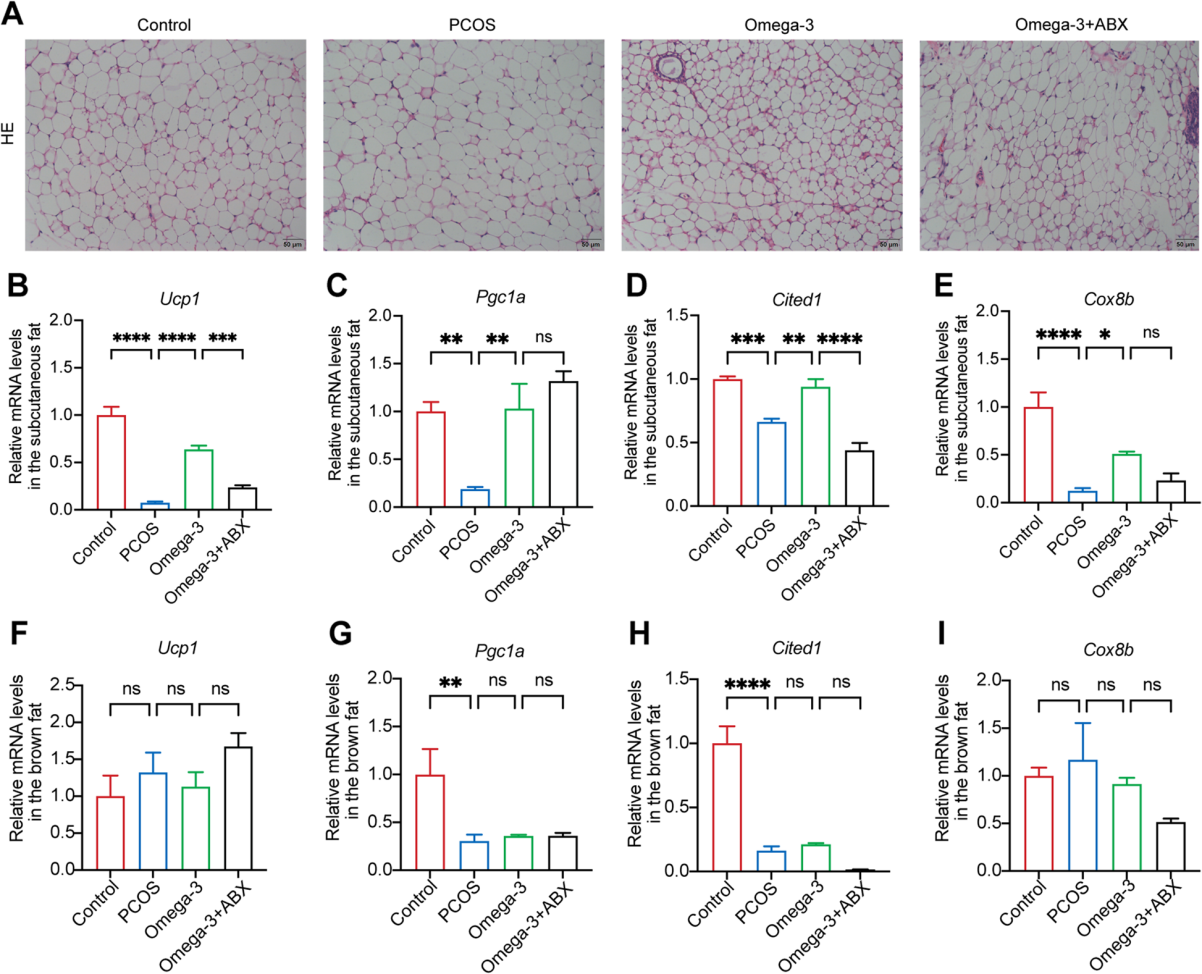
Omega-3 PUFAs improve the function and morphology of adipose tissues of PCOS in a gut microbiota–dependent manner. **A** Representative images of subcutaneous adipose tissues from control, PCOS, omega-3, and omega-3 + ABX mice. Scale bars: 50 μm. **B**-**E** Thermogenic markers in subcutaneous adipose tissues. **F**-**I** Thermogenic markers in brown adipose tissues. Error bars are expressed as the means ± SEMs. Statistical significance was determined by *P* values determined by one-way ANOVA followed by Dunnett’s multiple comparisons test (*P < 0.05; **P < 0.01; ***P < 0.001; ****P < 0.0001)

**Fig. 7:**
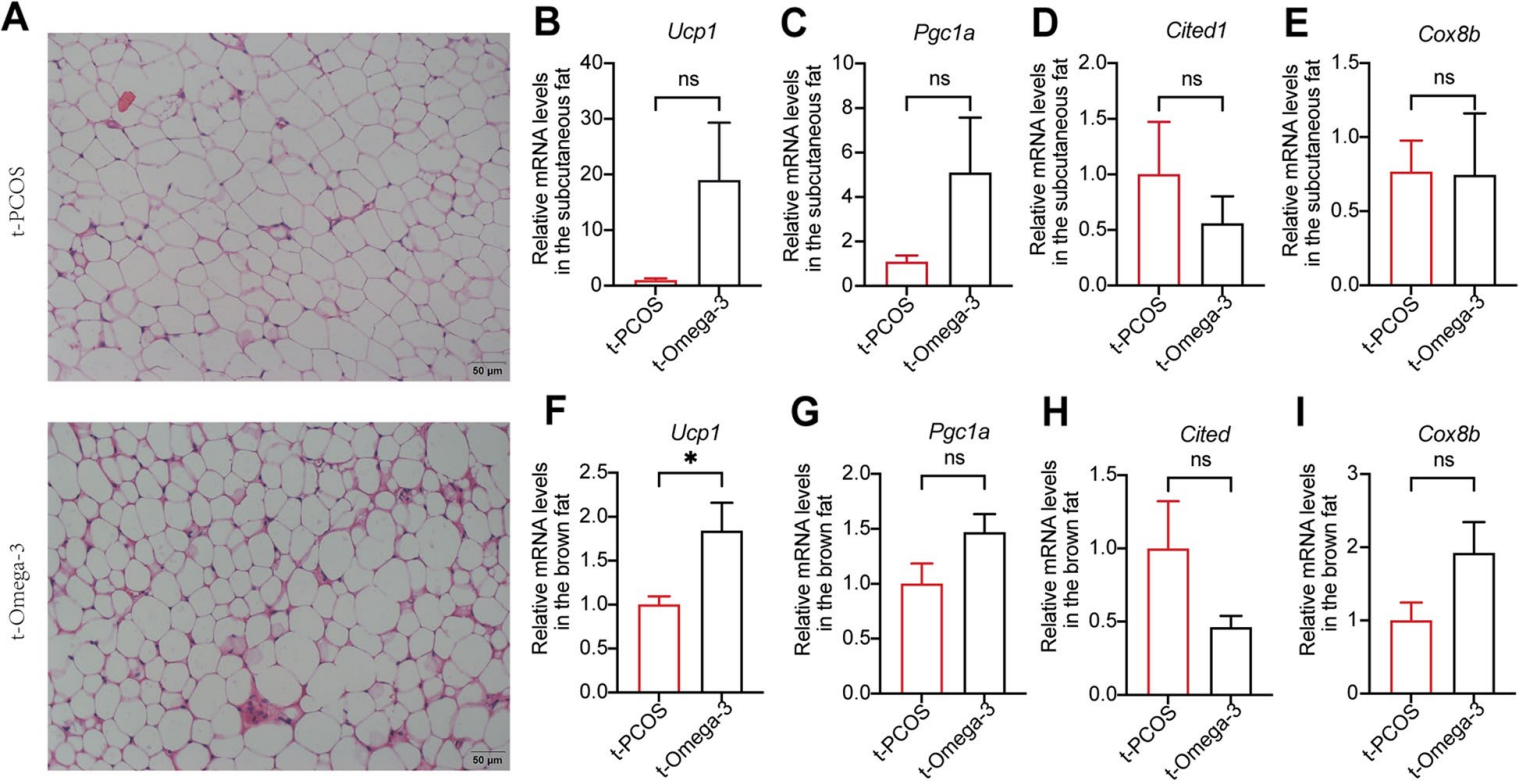
Transferring feces from omega-3 PUFA-treated mice to PCOS mice changed the function and morphology of adipose tissues. **A** Representative images of subcutaneous adipose tissues from t-PCOS and t-Omega-3 mice. Scale bars: 50 μm. **B**-**E** Thermogenic markers in subcutaneous adipose tissues. **F**-**I** Thermogenic markers in brown adipose tissues. Error bars are expressed as the means ± SEMs. Statistical significance was determined by *P* values determined by two-tailed paired-samples t test (*P < 0.05)

The possible mechanisms by which omega-3 regulates insulin resistance in PCOS were investigated. White adipose tissue browning increases the metabolic rate and improves insulin resistance [40, 41]. Transplantation of brown adipose tissue reversed anovulation, polycystic ovaries, insulin resistance and infertility in PCOS rats [42]. Therefore, the morphology of adipose tissues and thermogenic markers were measured in subcutaneous and brown adipose tissues from PCOS mice. Omega-3 PUFA-treated mice showed a decrease in multilocular cells within adipose tissues (Fig. 6A). PCOS mice exhibited significantly lower levels of Ucp1, Pgc1a, Cited1 and Cox8b mRNAs than controls in subcutaneous adipose tissues. Notably, the relative expression of these thermogenic markers in subcutaneous fat was dramatically elevated after omega-3 administration in the PCOS mice (Fig. 6B-E). However, although the expression of *Pgc1a* and *Cited1* in brown fat tissues was decreased in PCOS mice, omega-3 PUFA treatment did not change these thermogenic markers (Fig. 6F-I). Interestingly, different results were observed in omega-3 + ABX mice, that is, browning of adipose tissues was decreased, as revealed by the reduced expression of *Ucp1*, *Cited* and *Cox8b* in subcutaneous fat tissues and the increase in multilocular cells within the adipose tissues (Fig. 6A-E). However, transferring feces from omega-3 PUFA-treated mice to PCOS mice only increased *Ucp1* and *Pgc1a* expression in both subcutaneous and brown adipose tissues, and other thermogenic markers did not change significantly (Fig. 7A-I). These data indicated that the mechanisms underlying omega-3 PUFA-regulated improvements in the insulin resistance associated with PCOS likely involve effects on adipose tissue to decrease thermogenic markers and multilocular cells within the subcutaneous adipose tissues. Furthermore, the gut microbiota played a key role in the improvement of adipose tissue function and morphology by omega-3 in PCOS mice.

### Omega-3 PUFAs ameliorate ovarian dysfunction associated with PCOS and likely involve direct effects on inhibiting ovarian inflammation

**Fig. 8:**
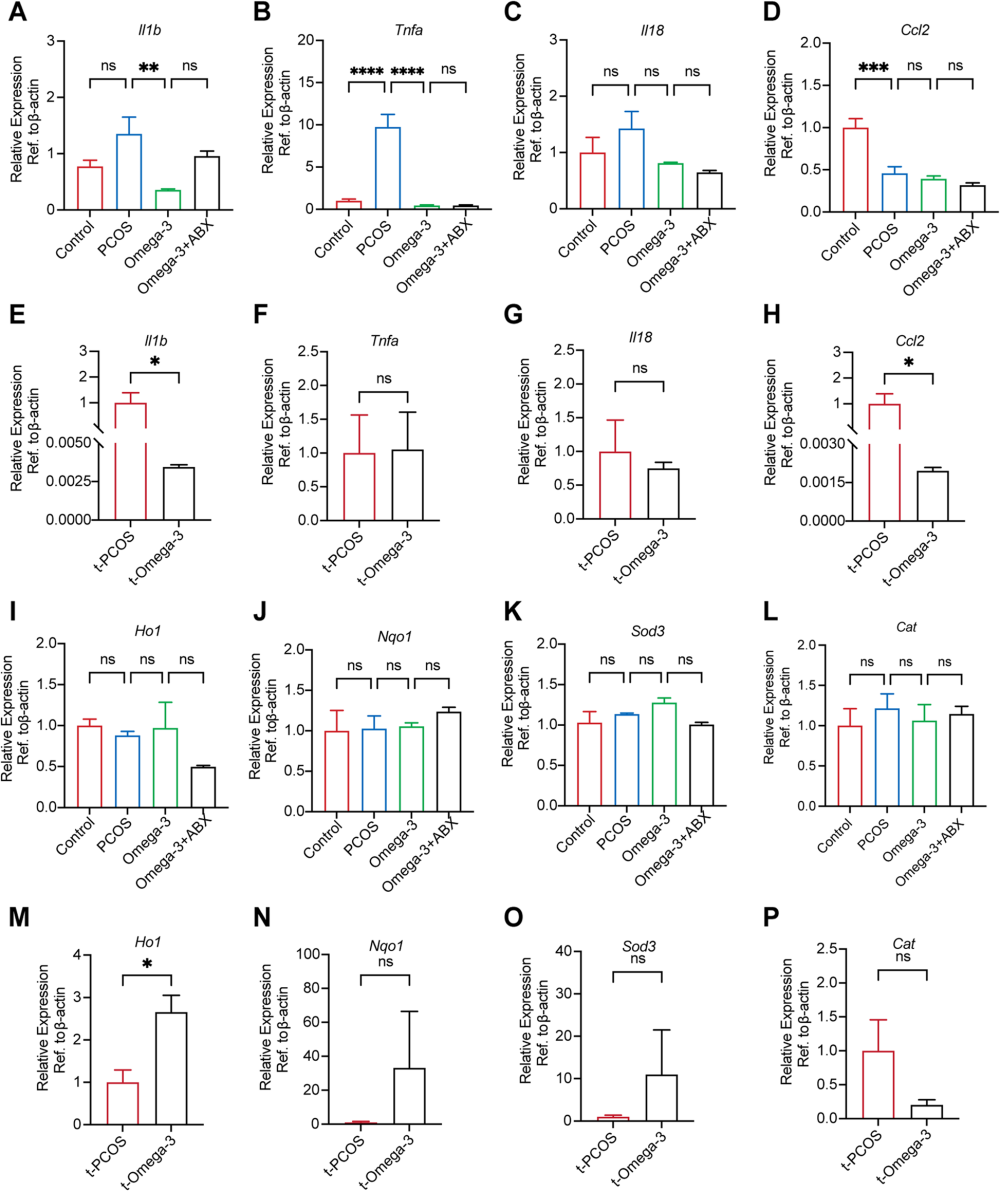
Omega-3 PUFAs ameliorate ovarian dysfunction associated with PCOS, likely involving direct effects on inhibiting ovarian inflammation. **A**-**D** The mRNA levels of IL-1β, TNF-α, IL-18 and CCL2 in the control, PCOS, omega-3 and omega-3 + ABX groups. **E**-**H** The mRNA levels of IL-1β, TNF-α, IL-18 and CCL2 in the t-PCOS and t-Omega-3 groups. Data are shown as the mean ± SEM. Statistical significance was determined by *P* values determined by one-way ANOVA followed by Dunnett’s multiple comparisons test and a two-tailed Mann–Whitney U test (*P < 0.05; **P < 0.01; ***P < 0.001; ****P < 0.0001)

The possible mechanisms by which omega-3 regulates ovarian function in PCOS were investigated. Inflammation and oxidative damage play an essential role in the pathogenesis of PCOS [43]. We next investigated whether omega-3 plays an anti-inflammatory role in the ovaries of PCOS mice. Supplementation with omega-3 PUFAs significantly reversed the enhanced expression of IL-1b, TNF-a and IL-18 mRNAs in PCOS mouse ovaries (Fig. 8A-C). However, the mRNA levels of CCL2 did not increase in PCOS mice (Fig. 8D). There was no difference between the omega-3 and omega-3 + ABX groups. Together, RT‒qPCR analyses confirmed that t-omega-3 mice showed decreased expression of *Il1b* and *Ccl2* (Fig. 8E, H). The mRNA levels of TNF-α did not change between t-PCOS and t-Omega-3 groups (Fig. 8F). There was a trend toward decreased *Il18* (Fig. 8G), but this difference was not statistically significant. Moreover, we investigated some antioxidants, such as *Ho1*, *Nqo1*, *Sod3* and *Cat.* However, they did not change (Fig. 8I-L). Transferring feces from omega-3 PUFA-treated mice to PCOS mice only increased *Ho1* expression in PCOS mouse ovaries, but other antioxidants did not change significantly (Fig. 8M-P). Our data indicated that the mechanisms underlying omega-3 PUFA-regulated improvements in the ovarian dysfunction associated with PCOS likely involve direct effects on the ovary to inhibit inflammation.

## Discussion

Although PCOS is a serious health concern worldwide, the limited treatment options primarily focus on the symptoms and therefore yield unsatisfactory therapeutic outcomes, and long-term metabolic complications such as obesity, type 2 diabetes mellitus (T2DM), and cardiovascular disease (CVD) exert a far-reaching influence on their quality of life [44]. Therefore, there remains an urgent need to develop effective therapies. Studies have shown that gut dysbiosis is characteristic of PCOS patients and rodent models. The manipulation of gut microbiota by FMT, probiotics, prebiotics or symbiotics ameliorates the PCOS phenotype [9]. FMT is a treatment that involves administration of a minimally manipulated microbial community from the stool of a healthy donor into the patient’s intestinal tract [45], which is a method to directly change the recipient’s gut microbiota to normalize the composition and gain a therapeutic benefit. Probiotics (e.g., *Lactobacillus*, *Bifidobacterium*, and *Streptococcus*) are live beneficial bacteria that, when ingested, may confer a health benefit on the host [46]. Prebiotics (e.g., lactulose and fructooligosaccharides, mainly inulin) are “nondigestible food ingredients that beneficially affect the host by selectively stimulating the growth and/or activity of one or a limited number of beneficial bacteria in the colon, and thus improve host health” [47]. Symbiotics are the combination of prebiotics and probiotics. It has been suggested that prebiotics, probiotics, and symbiotics could be a potential therapy for gastrointestinal disorders because of their beneficial effects on modifying the gut microflora [48]. As an anti-inflammatory and microbiota-regulating supplement, the potential effect of omega-3 PUFAs on PCOS is worth investigating. First, we conducted a detailed phenotypic assessment of PCOS and omega-3 PUFA-treated mice, and we found that omega-3 PUFA supplementation could ameliorate endocrine disorders, ovarian dysfunction and insulin resistance. Then, we showed that omega-3 PUFAs could alleviate gut dysbiosis in PCOS mice. The mechanisms underlying omega-3 benefits likely involve direct effects on the ovary to inhibit inflammation. Furthermore, the mechanism underlying omega-3 alleviation of IR involving the gut microbiota improved adipose tissue function and morphology by decreasing thermogenic markers and multilocular cells within the subcutaneous adipose tissues in DHEA-induced PCOS mice.

Omega-3 PUFAs are powerful antioxidative stress and anti-inflammatory agents. In addition, omega-3 PUFAs play a major role in male and female reproduction. Emerging evidence suggests that inflammation and oxidative stress contribute greatly to PCOS pathophysiology. In the present study, we observed that omega-3 PUFAs ameliorated estrous cycle disorders and ovarian morphology disruption and decreased serum testosterone and fasting blood glucose levels in DHEA-induced mice, which suggests that omega-3 PUFAs could improve ovarian dysfunction and insulin resistance in PCOS mice.

The gut microbiota is considered to be a full-fledged endocrine organ and plays an important role in the female reproductive endocrine system [49]. Growing evidence has demonstrated that the gut microbiota and its metabolites are closely associated with the occurrence and development of PCOS. Qiao X. et al. reported that *Bacteroides vulgatus* was markedly elevated in the gut of individuals with PCOS, and transplantation of fecal microbiota from women with PCOS or *B. vulgatus*-colonized recipient mice resulted in increased disruption of ovarian functions and infertility [9]. Liu et al. found a decrease in *Ruminococcaceae* in women with PCOS, which was consistent with our research [30]. α-diversity refers to the taxonomic diversity of bacteria present within a single sample [50]. α-diversity measures diversity within communities, and β-diversity measures diversity between communities. Here, we found that omega-3 PUFA treatment has an obvious effect on the α-diversity but not β-diversity of the gut microbiota in PCOS mice. In addition, omega-3 PUFA treatment displayed a decrease in the relative abundance of *Alloprevotella* and an increase in the relative abundance of *Akkermansia* and *Alistipes.* In general, these studies raised the possibility that *Akkermansia* might be important for the establishment of beneficial intestinal microbiota and could be developed into a probiotic therapy. However, whether *Akkermansia* could protect against ovary dysfunction was not determined in our study. It will be better to further replenish *Akkermansia* by oral gavage to accurately assess their effect. The gut microbiota affects host metabolism by interacting with host signaling pathways; therefore, further studies are required to elucidate the precise mechanism by which omega-3 PUFAs affect metabolism in PCOS mice. To our knowledge, the results of the current study suggest for the first time that omega-3 PUFAs can protect PCOS mice by improving white adipose tissue browning and are associated with the gut microbiota. Numerous studies have demonstrated that a chronic low-grade degree of inflammation plays a critical role in the development of PCOS. In our study, we indicated that omega-3 PUFA supplementation alleviated ovarian inflammation by suppressing the mRNA expression of IL-1b, TNF-a and IL-18 in PCOS mouse ovaries. MCP-1 (monocyte chemoattractant protein-1), also known as chemokine (CC-motif) ligand 2 (CCL2), is from the family of CC chemokines [51]. PCOS females have significantly increased levels of MCP-1 [14], and clinical studies have shown that supplementation with omega-3 PUFAs is beneficial in decreasing MCP-1 expression in macrophages and the levels of endothelial chemokines [52, 53]. However, the mRNA levels of CCL2 did not increase in PCOS mice in our study. Numerous studies have demonstrated that the protective effect of omega-3 PUFAs is associated with their ability to scavenge oxygen free radicals and reduce oxidative stress in target organs [54]. Interestingly, we observed no effect of omega-3 PUFA administration on the levels of *Ho1*, *Nqo1*, *Sod3* and *Cat* in the ovaries of DHEA-treated mice. Therefore, the protective effect of omega-3 PUFAs on DHEA-induced ovarian dysfunction was not attributed to reduced oxidative stress in ovaries. Our data indicate that the mechanisms underlying omega-3 PUFA-regulated improvements in the ovarian dysfunction associated with PCOS likely involve mainly direct effects on the ovary to inhibit inflammation. Ovarian granulosa cells (GCs) are critical in shaping follicular development. Evidence has also shown that the apoptosis rates of ovarian GCs in antral follicles in PCOS are significantly increased compared with those in healthy controls [55]. The follicles are comprised of three essential cells: theca cells, granulosa cells, and the oocyte. Together, these cells interact in a synergistic manner to secrete sex steroids and protein hormones that contribute to oocyte maturation for successful fertilization [56]. Gonadotropin-releasing hormone (GnRH) is released in a pulsatile fashion from the hypothalamus, inducing the release of the gonadotropins FSH and LH from the anterior pituitary gland. FSH acts directly on granulosa cells of the developing ovarian follicle to stimulate growth of the follicle, secretion of estrogen and expression of the LH receptor (LHCGR) [57–59]. LH stimulates androgen production by the theca cells of developing follicles and triggers ovulation when follicular maturation is complete [60]. During the ovulatory process, LH causes the final maturation and release of the ovum from preovulatory follicles and stimulates the differentiation of the theca and granulosa cells of the preovulatory follicle into the small and large steroidogenic cells of the corpus luteum, respectively [61, 62]. Defects in angiogenesis of the corpus luteum are at the origin of PCOS. In PCOS ovulatory cycles, infertility could result from a dysfunctional corpus luteum [63]. Recent research has focused on the function of the corpus luteum, theca cells and granulosa cells, which play a key role in ovarian androgen production and conversion. The specific cell molecular mechanisms underlying omega-3 PUFA-mediated improvement of PCOS ovarian functions remain to be further explored.

The findings that androgens can induce PCOS phenotypes and gut microbiota disturbance in rodents [64, 65] were validated in DHEA-induced PCOS-like mice. Clinical studies found that the gut microbial composition and relative abundance of taxa were related to PCOS phenotypes [66]. Zarrinpar et al. found that depletion of the microbiome by antibiotics reduced FBG levels and the GTT glucose surge in normal mice [67]. Han et al. found that the blood glucose curves showed similarly high levels in the DHEA and DHEA + antibiotics groups, suggesting that hyperandrogenism leads to glucose intolerance independent of the gut microbiome [44]. The clearance of gut microbiota did not avert polycystic morphology induced by DHEA, suggesting that the ovarian anomalies resulting from androgen administration were independent of the gut microbiota, which was consistent with our research. Here, we found that omega-3 PUFAs could alleviate gut dysbiosis in PCOS mice. When the gut microbiota was cleared with antibiotics, most of the protective effects of omega-3 PUFAs on ovarian dysfunction remained, and FMT showed few protective effects of colonized microflora on PCOS. These findings indicate that although omega-3 PUFAs can alter gut microbiota dysbiosis in PCOS mice, omega-3 PUFAs protect against endocrine disorders and ovarian dysfunctions in a gut microbiota-independent manner. However, the gut microbiota plays a key role in improving white adipose tissue browning.

## Conclusion

In conclusion, we found that omega-3 PUFA supplementation could ameliorate endocrine disorders, ovarian dysfunction and insulin resistance in PCOS mice. Omega-3 PUFAs could alleviate gut dysbiosis in PCOS mice. The mechanisms underlying omega-3 benefits likely involve direct effects on inhibiting ovarian inflammation. On the other hand, the mechanism underlying omega-3 alleviation of IR involving the gut microbiota improved adipose tissue function and morphology by decreasing thermogenic markers and multilocular cells within the subcutaneous adipose tissues in DHEA-induced PCOS mice.

## Supplementary Information

**Additional file 1.**

## Data Availability

The datasets supporting the conclusions of this article are included within the additional files.

## Abbreviations

PCOS: Polycystic ovary syndrome
PUFA: Polyunsaturated fatty acid
IR: Insulin resistance
DHEA: Dehydroepiandrosterone
16S rDNA: 16S ribosomal DNA
GCs: Granulosa cells
FMT: Fecal microbiota transplantation
SCFAs: Short-chain fatty acids
FSH: Follicle-stimulating hormone
LH: Luteinizing hormone
E2: Estradiol 2
T: Testosterone
GTT: Glucose tolerance test
ITT: Insulin tolerance test
PCA: Principal component analysis
UPGMA: Unweighted pair group method using arithmetic average
TNF-α: Tumor necrosis factor-α
IL-1β: Interleukin-1 beta
